# Cell cycle oscillations in a polarity network facilitate state switching by morphogenetic cues

**DOI:** 10.1101/2025.10.12.681824

**Published:** 2025-10-13

**Authors:** KangBo Ng, Hadjar Sebaa, Nisha Hirani, Alex Chizh, Zeno Messi, Tom Bland, Kenji Sugioka, Nathan W. Goehring

**Affiliations:** 1 The Francis Crick Institute, 1 Midland Road, NW1 1AT, London, UK; 2 Institute for the Physics of Living Systems, University College London, Gower St, WC1E 6BT, London, UK; 3 Centre for Developmental Neurobiology, King’s College London, London, UK; 4 Life Sciences Institute, The University of British Columbia, 2350 Health Sciences Mall, BC V6T1Z3, Vancouver, Canada; 5 Department of Zoology, The University of British Columbia, 2350 Health Sciences Mall, BC V6T1Z3, Vancouver, Canada; 6 Present address: Whitehead Institute for Biomedical Research, 455 Main St, MA 02142, Cambridge, USA; 7 Present address: Laboratory for Molecular Cell Biology (LMCB), University College London, Gower St, WC1E 6BT, London, UK; 8 Present address: Department of Molecular and Cellular Biology, University of Geneva, Quai Ernest-Ansermet 30, 1205, Genève, Switzerland

**Keywords:** Cell cycle, cell polarity, C. elegans, cell fate, robustness, oscillations, dynamic systems

## Abstract

The proper establishment of cell form, fate, and function during morphogenesis requires precise coordination between cell polarity and developmental cues. To achieve this, cells must establish polarity domains that are stable yet sensitive to guiding cues. Here we show that *C. elegans* germline blastomeres resolve this trade-off by creating a time-varying polarization landscape. Specifically, coupling the PAR polarity network to the cell-cycle kinase CDK-1 ensures that newborn cells operate in a low-feedback regime that lowers barriers to polarity state switching, allowing spatial cues to induce and orient PAR protein asymmetries. As CDK-1 activity rises at mitotic entry, increasing molecular feedback reinforces cue-induced asymmetries to yield robust and stable patterning of PAR domains. Consistent with this model, optogenetic and chemical perturbations show that low-CDK/low-feedback regimes destabilize PAR domains but are required for both *de novo* polarization and the reorientation of polarity in response to inductive cues. We propose that mitotic oscillations in cell polarity circuits dynamically optimize the polarization landscape to enable coordination of polarity with morphogenesis. Such temporal control of developmental networks is likely a general mechanism to balance robustness of cellular states with sensitivity to signal-induced state switching.

## Graphical Abstract

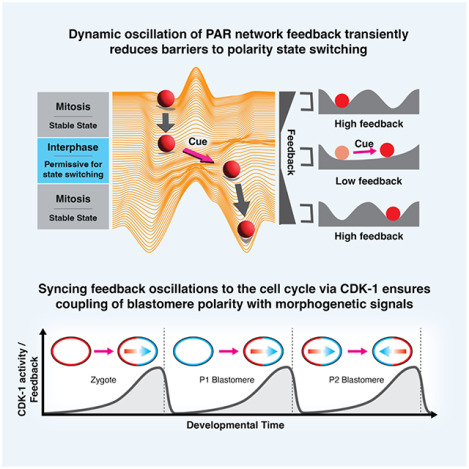

A key challenge for developmental signalling networks is to balance signal-sensitivity with output-stability. Robust signal responses are often attributed to feedback circuits that introduce bistable switch-like behavior or irreversibility into signaling networks ^1,2^. However, the same feedback that stabilizes outcomes against perturbations or stochastic variation can also reduce sensitivity, hindering the cells’ ability to respond to changes.

One context where this tradeoff is particularly evident is during cell polarization. Cell polarity describes the ability of cells to orient in space and typically involves self-organizing molecular networks which generate asymmetric protein patterns guided by spatiotemporal cues. One such network is the conserved PAR (*par*-titioning defective) network, which underlies a broad range of developmental processes in metazoans ^3-5^, including asymmetric cell division, cell migration, and organisation of tissue architecture. As the PAR network is continuously redeployed during development, it must remain sensitive to changing signals and cellular contexts so that polarity is properly oriented with respect to neighbouring cells or environmental cues. At the same time, once established, polarity must be sufficiently stable to reliably coordinate downstream processes in the face of perturbations. This dual requirement poses a paradox: cells that are too sensitive to signals will fail to maintain stable directionality, but introducing excessive stability into the network will hinder the ability of cells to respond and reorient with respect to spatial cues. Resolving this paradox is central to understanding how polarity is integrated into developmental programmes.

To address this question, we turned to the highly tractable *C. elegans* germline P lineage as a model. Beginning with the zygote (P0), P lineage blastomeres undergo a series of asymmetric divisions to generate the major founder lineages of the animal, including specification of the primordial germ cells ^6^. Importantly, these asymmetric divisions must be properly oriented by spatial cues so that the different cell types are correctly positioned within the embryo during development. This coordination requires that the polarity of P blastomeres adapt to dramatic shifts in cellular context, discriminate among competing cues, and respond to changing signals to polarize in the correct orientation ^3,6-12^; in other words, blastomeres must be highly sensitive to developmental cues, yet, once polarity is established it must be sufficiently stable to robustly inform downstream pathways, such as spindle position and segregation of fate determinants ^6^.

Despite blastomere-specific differences, the core principles of polarization are conserved. In each case polarization involves the cue-induced formation of two opposing, membrane-associated PAR domains, each harboring a distinct subset of PAR proteins termed anterior PARs (aPARs) ^13-17^ and posterior PARs (pPARs) ^18-21^, respectively. Mutual antagonistic feedback subsequently enforces segregation of these two PAR domains: the aPAR effector PKC-3 phosphorylates pPARs to exclude them from aPAR domains ^22,23^, whereas the pPAR effectors PAR-1 and CHIN-1 locally suppress aPARs within pPAR domains ^15,18,23-25^. Thus, comparative analysis of P blastomeres provides an ideal framework for dissecting how a single polarity network navigates the dual requirements for sensitivity and stability during development.

Here we reveal a central role for cell cycle-entrained oscillations in the PAR network in resolving this sensitivity-stability tradeoff. Examination of pPAR effectors reveals dynamic oscillation of their membrane localization in phase with CDK-1 activity. The resulting changes in network feedback shift blastomeres from a rheostat-like, low-feedback state early in the cell cycle, during which network behavior is highly responsive to instructive cues, to a high feedback, switch-like state that enforces stable patterns as the blastomere enters mitosis. We suggest that the pervasive mitotic oscillations in PAR protein behavior observed across metazoa ^26,27^ may reflect an ancestral design principle that allows cells to achieve both the signal-sensitivity and output-stability required for robust coordination of cell polarity with morphogenesis.

## Results:

### CDK-1 drives oscillations in pPAR effector membrane association

**Figure 1.**
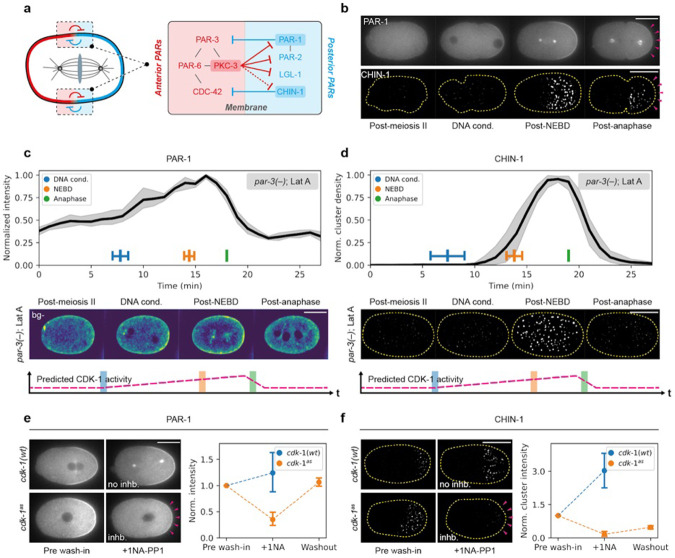
CDK-1 coupled oscillations in PAR behavior. **a,** A schematic illustrating mutual antagonistic feedback between aPARs (red) and pPARs (blue). For aPARs, the main effector is PKC-3, which locally removes all pPARs from the anterior membrane in the zygote. For pPARs, there are two effectors, PAR-1 and CHIN-1, which locally suppress PAR-3 and active CDC-42 from the posterior respectively. **b,** pPAR effectors exhibit oscillations in membrane levels over the cell cycle. Top, a time series of midplane confocal images of embryos expressing PAR-1::GFP with mCherry::PAR-2 (not shown) and H2B::mCherry in a *par-3(+/−)* background (NWG0434, roller phenotype) (n=4). Bottom, a time series of background subtracted (see Methods) cortical images of embryos expressing mNG::CHIN-1, H2B::mCherry and NMY-2::mKate2 (not shown) (NWG0528) (n=8). Magenta arrowheads indicate a sharp decrease in membrane levels following anaphase onset. Quantified (as in **c** and **d** in Supplementary Fig 1). **c,** Quantifications and images of embryos expressing PAR-1::GFP with mCherry::PAR-2 (not shown) and H2B::mCherry in a *par-3(−)* background (NWG0434, roller phenotype) treated with Latrunculin A throughout the cell cycle (n=5). Bottom shows the expected CDK-1 activity during the different cell cycle stages, which appears positively correlated with PAR-1 membrane levels. Times are normalized relative to anaphase. **d,** Same as **c**, but for embryos expressing mNG::CHIN-1, H2B::mCherry, NMY-2::mKate in a *par-3(−)* background (NWG0543) treated with Latrunculin A (n=5). Images in both **c** and **d** are background subtracted. **e,** PAR-1 membrane levels respond to changes in CDK-1 activity. Left, midsection confocal images of embryos expressing PAR-1::GFP and mCherry::PAR-2 (not shown) in either a *cdk-1(wt)* (NWG0042) (n=5) or *cdk-1*^*as*^ background (NWG0520) (n=7), acutely treated with 20μM 1NA-PP1 after pronuclear meeting (PNM). Note the reduction in PAR-1 membrane levels after CDK-1 inhibition (magenta arrowheads). Middle, the corresponding quantification for conditions shown on the left, showing also that PAR-1 membrane levels increase when CDK-1 inhibition is relieved. **f,** CHIN-1 membrane levels respond to changes in CDK-1 activity. Same as **e** but for cortical images of embryos expressing mNG::CHIN-1 in either a *cdk-1(wt)* (NWG0451) (n=4) or *cdk-1*^*as*^ background (NWG0518) (n=4). Mean and 95% confidence interval (bootstrapped) indicated. Scale bars, 20μm.

The two key pPAR effectors, PAR-1 and CHIN-1, have been reported to exhibit changes in membrane concentration throughout the zygotic cell cycle ^15,20^. Quantifications of PAR-1 and CHIN-1 membrane levels with respect to a cell cycle marker (Histone H2B) revealed tight coupling to mitotic transitions: membrane concentrations were initially low, first increased following chromosome condensation, peaked around nuclear envelope breakdown (NEBD), and then declined at anaphase onset (Fig. 1b-d, Supplementary Fig. 1). This pattern of oscillation was repeated in the P1 blastomere, suggesting P lineage blastomere divisions are characterized by repeated cycles of membrane association and dissociation of pPAR effectors (Supplementary Fig. 1).

We next sought to determine whether the oscillatory membrane association of pPAR effectors reflects direct coupling to the cell cycle, or regulation by other cell cycle-dependent processes, such as changes in the activity or localization of aPAR proteins, remodeling of the cortical actin cytoskeleton, centrosome growth cycles, or changes in the activity of key PAR regulators LKBP/PAR-4 or 14-3-3/PAR-5, could all contribute to membrane oscillations of pPAR effectors ^15,17,23,25,28-31^. However, consistent with intrinsic cell-cycle-dependent regulation, we found that oscillations persisted in *par-3(−)* embryos, in *par-3(−)* embryos treated with latrunculin A, and in embryos subject to RNAi targeting other PAR proteins (*par-2, par-4, and par-5*) or the core centrosome component *spd-5* (Fig. 1b-d, Supplementary Figs. 1-3).

The pattern of pPAR effector oscillations - low early in the cell cycle, increasing rapidly after mitotic entry, and declining at anaphase onset - roughly mirrored the expected activity of the mitotic kinase CDK-1 ^32-34^. To explicitly test whether pPAR membrane concentrations were controlled by CDK-1, we generated an analog-sensitive *cdk-1* (*cdk-1* ^*as*^) allele ^35-38^, allowing specific and acute inhibition of CDK-1 activity (Supplementary Fig. 4). Acute inhibition of CDK-1^AS^ with the ATP analogue 1NA-PP1 reduced PAR-1 and CHIN-1 membrane signals, which were partially restored upon drug washout (Fig. 1e,f, Supplementary Videos 1,2). Finally, we confirmed that changes in PAR-1 and CHIN-1 membrane levels were not due to changes in aPAR activity (Supplementary Fig. 4), strongly suggesting that pPAR effector oscillations are entrained by CDK-1 activity.

### CDK-1 drives oscillations between low and high feedback states

**Figure 2.**
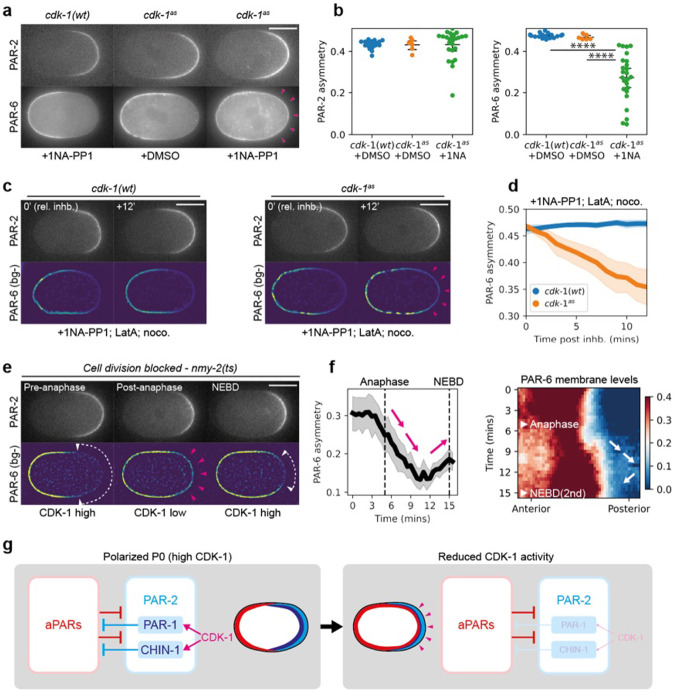
CDK-1 couples PAR feedback strength to the cell cycle. **a,** CDK-1 inhibition leads to reduction in PAR-6 asymmetry. Midsection confocal images of embryos expressing mCherry::PAR-2 and PAR-6::mNG in *cdk-1(wt)* (NWG0268) or *cdk-1*^*as*^ (NWG0443) background, treated with either DMSO or 50μM 1NA-PP1. Sample sizes: *cdk-1(wt)* + 1NA-PP1 (n=18), *cdk-1*^*as*^ + DMSO (n=8), *cdk-1*^*as*^ + 1NA-PP1 (n=25). Open magenta arrowheads indicate the presence of PAR-6 in PAR-2 occupied domains. **b,** Quantification of asymmetry for embryos corresponding to conditions shown in **a**. Each point represents data from a single embryo. **c,** PAR-6 invasion phenotype in CDK-1 inhibited embryos are not dependent on cytoskeleton. A time series of midplane confocal images, before and after combination treatment with 1NA-PP1, Nocodazole and Latrunculin A, for embryos expressing mCherry::PAR-2 and PAR-6::mNG in either a *cdk-1(wt)* (NWG0268) (n=6) or *cdk-1*^*as*^ (NWG0443) (n=7) background. Magenta arrowheads indicate PAR-6 invasion into posterior PAR-2 domains. PAR-6 images were background subtracted (bg-) to improve visibility due to poor signal-to-noise ratios. **d,** Quantification of PAR-6 asymmetry over time for conditions corresponding to **c**. **e,** PAR-6 invasion into PAR-2 domains is correlated with cell cycle stage. A time series of midsection confocal images of an embryo expressing mCherry::PAR-2 and PAR-6::mNG in an *nmy-2(ts)* background (NWG0509) (n=8). Acute temperature upshift to the restrictive temperature was performed to inhibit *nmy-2(ts)* activity after zygote polarization, decoupling cell division from cell cycle progression. White arrowheads with dotted lines indicate PAR-6 exclusion from PAR-2 domains. Open magenta arrowheads indicate the presence of PAR-6 in PAR-2 occupied domains. **f,** Quantification of PAR-6 asymmetry and membrane distribution over time for conditions corresponding to **e**. Magenta and white arrows both indicate loss of PAR-6 asymmetry following anaphase and increase in asymmetry before NEBD, corresponding to a presumptive loss and gain of CDK-1 activity respectively. **g,** Schematic showing that CDK-1 positively regulates P→A feedback via controlling PAR-1 and CHIN-1 membrane levels, leading to changes in the ability for PAR-2 domains to exclude aPARs. Mean and 95% confidence interval (bootstrapped) indicated. Scale bars, 20μm. Student’s t-test was performed, unpaired, two-tailed. Scale bars, 20μm. n.s. = not significant, *, p-value < 0.05, **, p-value < 0.005, *** p-value < 0.0005, **** p-value <0.00005.

To assess whether these CDK-1-dependent changes in pPAR effector membrane association correlated with changes in the ability of pPARs to antagonize aPARs ^23,25,39-41^, i.e. pPAR to aPAR (P→A) feedback, we examined the response of the aPAR protein PAR-6 to CDK-1 inhibition. Normally, PAR-6 is excluded by PAR-1 and CHIN-1 from the pPAR domain following polarization ^23^. However, upon inhibition of CDK-1, PAR-6 invaded the posterior domain as marked by PAR-2, leading to overlap of the two proteins at the posterior (Fig. 2a,b, Supplementary Fig. 5a). This suggests that CDK-1 inhibition reduces P→A feedback. This phenotype did not appear to stem from off-target effects on the cytoskeleton or regulators downstream of CDK-1 such as AIR-1 and PLK-1, which have also been implicated in PAR polarization (Fig. 2c,d, Supplementary Fig. 5, Supplementary Video 3) ^42-47^.

We next looked at the response of PAR-6 to oscillations in CDK-1 activity through the cell cycle. In wild-type zygotes, we observed posterior spreading of PAR-6 following anaphase onset, when CDK-1 activity and P→A feedback is expected to be low (Supplementary Fig. 6a,b). However, we are unable to accurately quantify the full extent of the spreading, as formation of the cleavage furrow is able to alter the positioning of PAR proteins ^11,48^. To circumvent this, we inhibited cytokinesis via acute inactivation of NMY-2 using a temperature sensitive *nmy-2(ts)* allele ^49^, or by depleting the formin CYK-1 ^50,51^. In both cases, PAR-6 transiently invaded the posterior PAR-2 domain following anaphase onset and was then cleared from the posterior as the embryos re-entered mitosis (~NEBD) of the second cell cycle (Fig. 2e,f, Supplementary Fig. 6c).

Consistent with this result, we observed cycles of aPAR-pPAR overlap and clearance in later P blastomeres P1-P3 as previously described ^11,12^. Specifically, PAR-6 accumulated steadily throughout the membrane early in the cell cycle, despite these cells being born with uniformly high membrane levels of PAR-2 (Supplementary Fig. 6d,e). PAR-6 only cleared from PAR-2 domains as the cells entered mitosis, resulting in mutually exclusive polarity domains.

Detecting reduced pPAR to aPAR feedback early in the zygotic (P0) cell cycle is complicated by the fact that pPARs are initially absent from the membrane following meiosis II due to the high initial levels of aPARs at the plasma membrane ^46^. However, by partially depleting PAR-6, we were able to generate zygotes in which pPARs were uniformly enriched on the plasma membrane (Supplementary Fig. 6f). In these zygotes, the residual PAR-6 on the plasma membrane initially overlapped with PAR-2 early in the cell cycle, but was rapidly cleared at mitotic entry (NEBD). Altogether, our data indicates that P→A feedback oscillates in phase with CDK-1 activity, shifting from low feedback early in the cell cycle to high feedback late (Fig. 2g,h).

### Oscillatory feedback allows robust response to polarizing cues from diverse initial states

We next investigated the potential impact of this oscillation between low and high feedback regimes by turning to a simplified two component reaction-diffusion model for polarity (see Methods) ^8,9,53^. As described previously, this system relies on interconversion between membrane-associated and cytoplasmic states, limiting pools, mass conservation and mutually antagonistic feedback. Previous work has shown that for high, balanced levels of feedback, the system is multistable, capable of supporting both uniform and polarized states ^8,52^.

**Figure 3.**
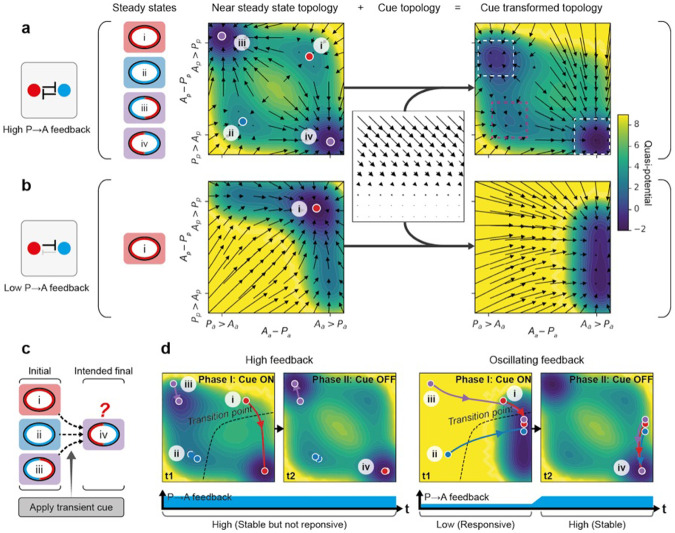
Oscillating feedback facilitates robust and cue-responsive polarization from diverse initial states **a,** High feedback produces stable attractors for polarity states but is unable to respond to cues from a range of initial states. A balanced high feedback between aPARs (A) and pPARs (P) facilitates 4 possible steady states. An unstable steady state at the center of the diagram is not shown ^8,52^. State i - uniform A high, ii - uniform P high, iii - polarized with pPARs high at the anterior and aPARs high at the posterior, iv - polarized with aPARs high at anterior and pPARs high at posterior. These 4 steady states can be mapped onto a concentration-difference landscape, defined by the difference between A and P at the anterior (A_a_ - P_a_) and the difference between A and P at the posterior (A_p_ - P_p_). Quivers represent the directionality and velocity of movement towards each of these steady states for every system state in the landscape. System behavior under cue induction can also be visualised by transforming the landscape with the cue topology, which is represented as a fractional redistribution of aPARs from the anterior to posterior (A_p_ to A_a_) continually, inducing polarization towards state iv. In the case of high P→A feedback, there is a region near state ii and iii in the landscape that can not respond well to cue induction. Quasi-potential is defined as the -ln(probability) that a stochastic simulation, started from a random initial condition near the system’s steady state, arrives at a given position in the phase plane. We performed 10⁷ independent simulations to estimate this quantity (see Modelling Supplement). As the full system is four-dimensional (A_a_, A_p_, P_a_, P_p_), but we project and visualize the results in two-dimensions (A_a_ - P_a_, A_p_ - P_p_), the resulting “potential-like” surface averages over behaviours that differ along the hidden dimensions, which could lead to mismatch with the quivers. Nonetheless, the quasi-potential still conveys the relative stability of each state in the landscape, in the presence of noise. To confirm that these behaviors are similarly present in a non-dimensional-reduced model, we repeated the simulations in a single species polarity model, which is fully tractable; we observed that feedback can similarly act against the cue topology to prevent accurate polarization (Supplementary Fig. 9). Dotted white boxes indicate steady state attractors of the system, whereas the magenta box indicates transient trapping of the system in a shallow well. **b,** Low feedback does not support stable polarized states, but promotes cue-responsiveness. Here, there is only one stable state, which is state i - uniform A high. However, in presence of a cue, all points across the landscape are correctly redirected towards the state iv quadrant, unlike in the case of high feedback. **c,** Schematic illustrating the full simulation of PAR polarization related to **d**, intending to polarize systems from state i, ii and iii to state iv using the same cue described above. **d,** Oscillatory feedback allows stable yet adaptable polarization from all points in the landscape. Left, in the constant high feedback condition, only points around state i are able to respond reliably to cues and move beyond the transition point for polarization towards state iv. Right, in the oscillatory feedback condition, temporary low feedback facilitates cue-responsiveness towards the transition point for all initial states, before increasing feedback locks in the stable polarized state. Importantly, all these properties are similarly captured in a full partial differential equation model, validating our approach (Supplementary Fig. 10).

To comprehensively capture system behavior during polarization, we constructed a landscape of different polarity states, described by the concentration difference between aPARs (A) and pPARs (P) in the anterior (A_a_-P_a_) and in the posterior (A_p_-P_p_) (Fig. 3) ^54^. Consistent with the multistable behavior of the system, for any given initial state the system tends to evolve towards one of four steady-states that defines the four quadrants in the landscape: (i) uniform A high, (ii) uniform P high, (iii) PA polarized - P high in anterior, A high in posterior, (iv) AP polarized - A high in anterior, P high in posterior (Fig. 3a, Supplementary Fig. 7). Thus, high feedback creates well-defined wells or attractors, with polarization effectively represented by a transition between alternative polarity states. In the case of the zygote, polarization would reflect a transition between states (i) and (iv). Note that while we do not observe other potential transitions in the zygote (i.e. polarization from a uniform P high state (ii to iv) or polarity reversal (iii to iv)), such transitions are observed in later P blastomeres ^7,11,12^ (see below).

In early *C. elegans* embryos, cues are thought to operate via local depletion or inhibition of aPAR activity ^8,11,12,22,25,55-57^. To represent these cues generically, we implemented a continuous process of fractional redistribution of A from posterior to anterior. When transformed with this cue, we found that the uniform A high state (i) vanishes and initial states in this quadrant evolve towards the AP polarized state (iv). Thus, consistent with prior analysis, this high feedback regime effectively captures polarization of the zygote. By contrast, initial uniform P (ii) and reversed PA polarity states (iii) were more resistant to the cue, with the system remaining trapped near the initial state. Thus, for fixed high feedback and an aPAR-acting cue, proper cue-oriented polarization is only observed for systems beginning in an initially uniform A state.

We next considered a low feedback regime in which P→A feedback is reduced (Fig. 3b). For sufficiently low P→A feedback, attractors states reflecting uniform P high (ii) and polarized states (iii, iv) vanish, leaving only a single uniform A high attractor (Supplementary Fig. 7). Thus, no stable polarized states are possible. Transforming this low feedback landscape with the cue shifted the single attractor towards the AP polarized quadrant. Thus, in this regime, all initial states converge on a single state exhibiting properly oriented AP polarity (Supplementary Fig. 7). However, when the cue is removed, the attractor reverts to a uniform A state and asymmetry is lost. Thus, while a low feedback regime is optimized for ensuring proper cue-induced asymmetry from divergent initial states, the resulting asymmetry is unstable.

Finally, we considered an oscillatory feedback regime in which cue induction is accompanied by a transient reduction in P→A feedback, similar to what is observed in embryos, where the low feedback state coincides with the time at which symmetry-breaking cues are active (Fig. 3c-e, Supplementary Videos 4,5). In this regime, all initial states converge towards a single attractor during the low feedback, cue “on” phase, before evolving towards the AP polarized state (iv) as the system returns to the initial high feedback, cue “off” regime. Thus, by transiently destabilizing the stable attractor states, oscillatory feedback drives convergence of the system from divergent initial states towards a single, stable polarity outcome. For completeness, we examined the behavior of the system when both P→A and A→P feedback were reduced simultaneously (Supplementary Fig. 8). The results were qualitatively similar, confirming the general benefit feedback oscillations have for promoting responsiveness of patterning systems to spatial cues. Specifically, by destabilizing potential attractors that might otherwise trap the system, oscillatory excursions into low feedback regimes provide transient windows of responsiveness to signal induced changes in state.

Because of the selective nature of the polarity cue in early *C. elegans* embryos, polarization from a uniform A state (e.g. the zygote) is not dependent on oscillation in P→A feedback. However, these results predict that oscillations through a transient low feedback state become critical in two situations: (1) When blastomeres must polarize from a uniform P state or (2) when blastomeres must reverse or reorient a pre-existing polarized state. As we show below, the P blastomeres P1 and P2, respectively, provide clear examples of these two cases, and thus provide an ideal opportunity to test these predictions ^7,11,12,58^.

### Oscillatory feedback facilitates correct response to polarity cues

Polarization of the P blastomeres, P1 and P2, begins with pPARs uniformly occupying the membrane while aPARs remain largely cytoplasmic ^6^. In both cells, an early cue drives pPAR polarization toward the embryo posterior. This cue is linked to a modest enrichment of aPARs at the nascent cell contact site induced by cortical flows during cytokinesis ^11^. However, the two cells diverge in their subsequent polarization patterns. P1 cells possess at least two semi-redundant pathways that reinforce this initial asymmetry ^11,12^, stabilizing polarization of aPARs at the anterior and pPARs at the posterior. In contrast, P2 must override and reorient this initial polarity domain in response to a signal from its sister cell EMS ^7^. This second cue effectively reverses the PAR domains ^58^, leading to enrichment of pPARs at the anterior and aPARs at the posterior. Thus, P1 and P2 reflect ideal test cases for examining whether cue-driven polarization from pPAR high states and polarity reversal, respectively, require a transient period of reduced feedback.

**Figure 4.**
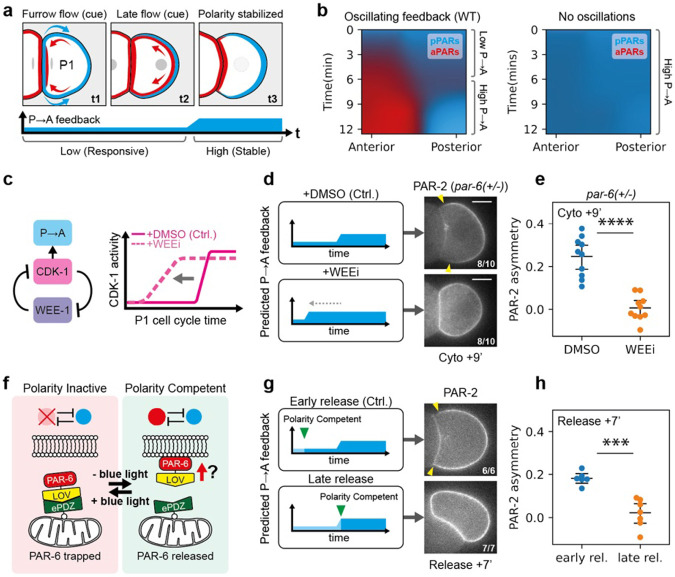
Transient reduction in feedback is required for robust polarization of P1 blastomeres. **a,** Schematic illustrating PAR polarization in P1, alongside the expected dynamics in P→A feedback throughout the cell cycle. **b,** Simulations of a modified PAR model representing P1 polarization suggests a requirement for oscillatory feedback (see Supplementary Fig. 11,12). **c,** Schematic illustrating regulation between WEE-1, CDK-1 and P→A feedback. Inhibiting the negative regulator of CDK-1, WEE-1 leads to precocious CDK-1 activation, and could shorten the low P→A feedback period. **d,** Left, schematic showing the expected shortening of P→A feedback in acutely WEE-1 inhibited embryos (WEEi; PD0166825) relative to control DMSO treated embryos. Right, representative midplane confocal images of an embryo expressing GFP::PAR-2 in a *par-6(+/−)* background (NWG0323), acutely treated with either DMSO (n=10) or 20μM WEEi (PD0166825) (n=10). Yellow arrowheads indicate the extent of the polarized PAR-2 domain. **e**, the corresponding quantification of PAR-2 asymmetry (ASI) 9 minutes after cell birth. **f,** Schematic illustrating the optogenetic knocksideways approach to trap PAR-6. In the presence of blue light, PAR-6 is sequestered at the mitochondria, preventing access to plasma membrane binding and thus polarity competence. In absence of blue light, PAR-6 is now free to bind to the membrane, making the system competent to polarization. **g,** Left, schematic showing when the system is made polarity competent relative to the dynamic P→A feedback cycles, comparing between early release, where the system is made polarity competent during the period of low P→A feedback, or late release, where the system is made polarity competent after P→A feedback is high. Right, representative midplane confocal images of embryos expressing PAR-6::GFP::LOV, TOMM-20::ePDZ::Halo and GFP::PAR-2 (NWG0597), with mitochondria-trapped PAR-6 released early (~3mins) (n=6) or late (~7.5mins) (n=7) after cell birth. Yellow arrowheads indicate the extent of the polarized PAR-2 domain. **h**, the corresponding quantification of PAR-2 asymmetry (ASI) 7 minutes after PAR-6 release.

We first focused on P1 polarization as a model for polarization from a uniform pPAR initial state (Fig. 4a,b, Supplementary Fig. 11,12). While we were unable to completely eliminate the low feedback state early in the cell cycle, we could significantly truncate it through inhibition of the CDK-1 inhibitor WEE-1 (Fig. 4c) ^59,59-62^. Consistent with premature CDK-1 activation, when we inhibited WEE-1, embryos exhibited accelerated loading of PAR-1 and CHIN-1 onto the membrane as well as precocious removal of PAR-6 from PAR-2 enriched membranes (Supplementary Fig. 13). Importantly, both effects were blocked by concomitant inhibition of CDK-1, confirming specificity of the effects on WEE-1 inhibition. These phenotypes were also unaffected by latrunculin A, ruling out contributions from altered cortical actomyosin reorganization that may ectopically induce asymmetries. In otherwise wild-type embryos, we found that WEE-1 inhibition did not prevent polarization, suggesting the initial, albeit shortened, period of low feedback was sufficient in these cases (Supplementary Fig. 13). However, WEE-1 inhibition rendered embryos sensitive to even modest reductions in aPAR levels. Specifically, when we inhibited WEE-1 in heterozygote *par-6(+/−)* embryos, which otherwise polarize normally, polarization was strongly compromised as scored either by qualitative or quantitative measures of PAR-2 asymmetry (Fig. 4d,e).

As an alternative to truncating the low feedback period, we implemented an optogenetic knocksideways approach (Fig. 4f, Supplementary Fig. 14, Supplementary Video 6) ^63,64^. By transiently sequestering PAR-6 at the mitochondrial surface, we could delay when the PAR network becomes competent for polarization and thereby explicitly test whether polarization of P1 must be initiated during the low feedback state. We found that early release during the low feedback period resulted in rapid PAR-6 loading and eventual PAR-2 polarization (Fig. 4g,h, Supplementary Fig. 14). By contrast, when we delayed release to a time that corresponded to onset of the high feedback state, PAR-6 was effectively locked out of the membrane and PAR-2 polarization failed.

**Figure 5.**
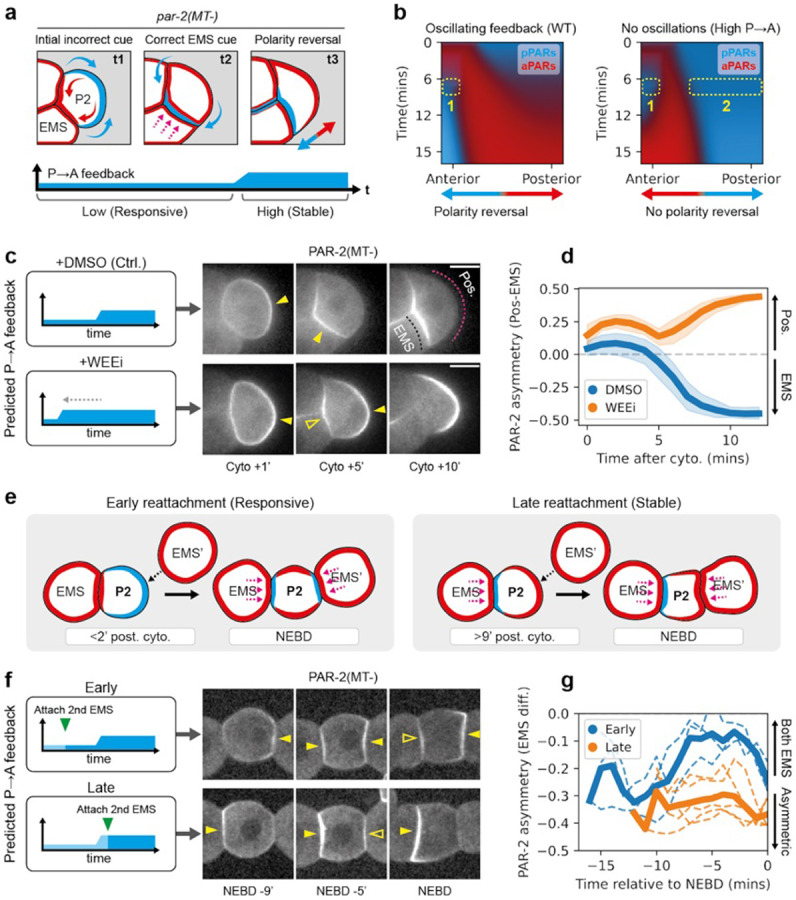
Cue-induced polarity reversal in P2 blastomeres requires a low feedback state. **a,** Schematic illustrating PAR polarization in P2 alongside the expected dynamics in P→A feedback throughout the cell cycle. P2 experiences two distinct cues, an initial incorrect cue that positions aPARs towards the anterior contact site and pPARs away towards the posterior. This is followed by a second cue provided by EMS, which recruits pPARs in the correct direction towards the anterior, effectively reversing the polarity induced by the initial cue. **b,** Simulations of a modified PAR model representing P2 polarization suggests a requirement for oscillatory feedback. Note that the oscillating and constant high feedback system behave differently when the second cue is introduced, while the former exhibits only one pPAR domain at the anterior while the posterior pPAR domain dissipates (dotted rectangles), the latter exhibits two pPAR domains at both the anterior and posterior end respectively. **c,** Left, predicted feedback dynamics in control or WEE-1 inhibited conditions. Right, time series of midsection confocal images of embryos expressing GFP::PAR-2(MT−) and PAR-6::mScarlet-I (NWG0639) (PAR-6 shown in Supplementary Fig. 15), treated with either DMSO (n=9) or 20μM WEEi (n=8). Yellow closed arrowheads indicate polarized PAR-2(MT−) domains, while yellow open arrowheads indicate the appearance of a second smaller PAR-2(MT−) domain. Magenta and black dotted lines indicate the region used for quantifying PAR-2 at the EMS-contact or embryo posterior in **d**. **d,** PAR-2 membrane asymmetry over time between the EMS-contact and embryo posterior after cytokinesis completion (cell birth) for DMSO vs WEEi treated embryos. Mean and 95% confidence interval (bootstrapped) indicated. **e,** Schematic illustrating embryo attachment experiments. Dissected EMS-P2 cell pairs were attached with a second ectopic EMS, EMS’, either early in the cell cycle or late. **f,** Left, predicted feedback levels in P2, when the ectopic EMS’ is attached early or late. Right, time series of midsection confocal images of dissected embryos expressing GFP::PAR-2(MT−) (NWG0192) attached with an ectopic EMS’ early (n=3) or late (n=5). Yellow closed arrowheads indicate the presence of PAR-2(MT−) domains, while yellow open arrowheads indicate a second smaller PAR-2(MT−) domain. **g,** quantifications for conditions corresponding to **f**. Mean and results for individual experiments are shown, due to the variable cell cycle stage following completion of EMS’ attachment. Scale bars, 20μm.

Next, we used P2 as a model to investigate cue-induced polarity reversal (Fig. 5a,b). We again utilized WEE-1 inhibition to shorten the initial period of low feedback and performed the experiments in embryos harboring *par-2(MT−)* mutations ^25^, as polarity reversal is more obvious in this background (Fig. 5c,d) ^11^. Consistent with prior descriptions, control *par-2(MT−)* embryos (treated with DMSO) exhibited initial PAR-2 polarization away from the cell contact towards the posterior (Fig. 5c,d). This posterior enrichment then rapidly dissipated as PAR-2(MT−) was recruited to the EMS:P2 cell contact. Ultimately, PAR-2(MT−) was consolidated to a single anterior domain in the anterior of P2. WEE-1-inhibited embryos also showed initial posterior enrichment; however, in contrast to control embryos, this posterior PAR-2(MT−) domain persisted even as PAR-2 was recruited to the anterior EMS-P2 contact, leading to the transient coexistence of two PAR-2 domains (Fig. 5c,d, Supplementary Video 7). Eventually, the anterior PAR-2(MT−) domain dissipated and was consolidated into the initial posterior domain. In other words, although the effects of the cue are clearly visible in recruiting PAR-2 to the EMS-P2 contact, it cannot outcompete the initial furrow-induced posterior domain, which appears to be locked in by the premature increase in feedback. Thus, polarity reversal fails.

To corroborate the WEE-1 inhibition experiments in P2, we turned to isolated blastomeres, which allowed us to control precisely when P2 received signals from EMS (Fig. 5e). Specifically, isolated EMS–P2 cell pairs were brought into contact with a second, ectopic EMS (EMS′), positioned opposite the native EMS, either early in the P2 cell cycle (0–2 min, when feedback is low) or late (9–11 min, when feedback is high) (Fig. 5f,g). In early P2 cells, PAR-2(MT−) exhibited a bias away from the native EMS contact, presumably due to furrow-induced asymmetry of aPARs. Attachment of an ectopic EMS′ to these early P2 cells typically produced PAR-2(MT−) domains of roughly equal size at both EMS contacts (ASI shifts towards 0), consistent with a robust polarization response to both cues (Fig. 5f,g, Supplementary Fig. 15). In late P2 cells, PAR-2(MT−) already showed a slight bias towards the native EMS contact. In this case, ectopic EMS′ attachment also generated an additional PAR-2(MT−) domain, consistent with P2 still receiving a signal (Supplementary Fig. 15). However, this secondary domain was unable to compete with the native domain: the domain was markedly weaker than the original and eventually dissipated, with PAR-2(MT−) ultimately consolidating at the native contact by NEBD. These findings are consistent with cue-sensitivity being limited to the early phase of the P2 cell cycle, which may explain previous reports that the ability of EMS to reorient pPAR domains was restricted to a narrow time window after birth of P2 ^7^.

Importantly, these behaviours were well captured by our model when modified to account for the particular features of P1 and P2, suggesting that changes in feedback dynamics alone are sufficient to account for the observed phenotypes (Fig. 4b, 5b, Supplementary Fig. 16-18).

Taken together, our data demonstrate that the cycling of the PAR network into a low feedback regime early in the cell cycle creates a critical cue-sensitive window that allows cells to properly polarize from diverse initial states and reorient polarity in response to directional cues.

## Discussion:

Coupling between cell polarity and the cell cycle has been observed in a broad range of biological systems from bacteria to humans ^26,27,37,46,65-73^. However, the role for such coupling is not clear. In fact, mitotic destabilization of polarity has been reported to sensitize proliferative epithelia to oncogenic transformation due the associated disruption of tissue integrity ^73^, raising the question of why transient destabilization is so conserved. Our data indicates that coupling of feedback in the PAR network to CDK-1 activity cycles the system between low and high feedback states, allowing temporal optimization of network activity. The resulting dynamic polarity landscape facilitates cellular responses to guiding cues by transiently reducing barriers to polarity state switching.

Combining experiments and theory, we show that high antagonistic feedback has opposing effects on cue-driven polarization. On one hand, strong feedback imposes a switch-like behavior in the system, locking in patterns and committing the cell to a specific polarity configuration. However, this comes at the cost of rendering embryos refractory to new cues. Oscillation of PAR activity resolves this conflict by transiently lowering feedback, allowing cells to enter a rheostat-like regime in which the network is highly sensitive to signals and asymmetries largely reflect spatial inputs. These asymmetries can then be reinforced and stabilized as feedback increases again. By temporally ordering periods of network sensitivity and stability, the introduction of cell cycle oscillations in network activity allows cells to sustain robust polarization, while providing for transient periods of cue-sensitivity that allow tight coordination between polarity and morphogenesis.

This paradigm of resolving conflict between cue-sensitivity and pattern stability via oscillations has roots in theoretical work on reaction-diffusion based models of cell polarity ^74-78^. Like the PAR system, these models often rely on stabilizing feedback to support stable polarization in response to weak, transient cues. However, self-stabilizing feedback similarly renders these polarity models resistant to reorientation by secondary cues, posing a problem for systems that must adapt to changing signals ^76,77,79^. One solution is to incorporate delayed negative feedback into the network, which renders polarity fronts self-limiting. Conceptually similar to what we describe in this study, the resulting periodic destabilization of polarity in these so-called excitable systems provides an opportunity to repolarize in a new direction. In chemotaxing cells, it is thought that such oscillatory behaviour balances exploration (sensitivity) and persistence (stability), enabling them to accurately track time-varying signals ^74,76,77,80^. Although these free-running oscillations are ideal for exploratory chemotaxing cells, it is less compatible for systems that need to strictly coordinate polarity behavior with developmental events. In these cases, such as the PAR system, entrainment of oscillations to a developmental controlled clock such as the cell cycle provides an attractive solution, as it allows coupling of the sensitivity of the system to guiding cues with developmental events, e.g. cell division.

The widespread prevalence of links between the cell cycle and cell polarity networks suggests that this paradigm of cell cycle-dependent feedback oscillations will be a common solution for balancing the stability of polarized states with their sensitivity to spatial cues, even if the precise timing and mechanisms are not strictly conserved. This includes stem cell systems such as neuroblasts which must coordinate polarity with niche signals ^37,69,81-84^, dividing epithelial cells which must ensure proper coordination of both apical basal and planar cell polarity with the surrounding tissue ^67,68,72,73,85-89^, and even budding yeast which must integrate spatial cues to ensure proper bud site selection ^90-92^. In each case, we speculate that low feedback regimes provide critical windows for priming cells to respond and adapt polarity guiding cues - in some cases effectively resetting polarity - before cells commit to a stable polarized state that is robust to perturbation.

While we have focused on changes in CDK-1-dependent regulation of pPAR to aPAR feedback, it is important to note that most PAR proteins exhibit some form of cell cycle dependent behavior ^15,23,26,37,81,93-95^, and we cannot rule out similar effects of feedback oscillations in aPARs. Both PAR-3 and CDC-42 behavior vary in a cell cycle dependent manner, though direct evidence for changes in aPAR to pPAR (A→P) feedback is currently lacking. Our theoretical analysis indicates that such coincident oscillation of both P→A and A→P feedback has similar effects on balancing stability and sensitivity, and, in fact, tends to further enhance and generalize the sensitivity of the PAR network to spatial cues, particularly to those that may not act via aPARs as we consider here.

Overall, we suggest that oscillatory signaling dynamics offer systems a way to temporally modulate network dynamics and thus satisfy the often paradoxical requirement for cells to exhibit sensitivity to signals and stability in outputs. This dynamical systems view of signalling network behavior giving rise to a time-varying polarization landscape, in this case entrained by the cell cycle, is particularly attractive for understanding how cells optimize their ability to sense, respond, and adapt to the continuously changing demands of developmental programmes.

## Supplementary Material

Supplement 1

Supplement 2

Supplement 3

Supplement 4

Supplement 5

Supplement 6

Supplement 7

Supplement 8

## References

[1] El-Samad H. Biological feedback control—Respect the loops. Cell Syst. 12, 477–487 (2021). 34139160 10.1016/j.cels.2021.05.004

[2] Freeman M. Feedback control of intercellular signalling in development. Nature 408, 313–319 (2000). 11099031 10.1038/35042500

[3] Lang C. F. & Munro E. The PAR proteins: from molecular circuits to dynamic self-stabilizing cell polarity. Development 144, 3405–3416 (2017). 28974638 10.1242/dev.139063 PMC5665476

[4] Buckley C. E. & St Johnston D. Apical–basal polarity and the control of epithelial form and function. Nat. Rev. Mol. Cell Biol. 1–19 (2022). 10.1038/s41580-022-00465-y 35440694

[5] Goldstein B. & Macara I. G. The PAR proteins: fundamental players in animal cell polarization. Dev. Cell 13, 609–622 (2007). 17981131 10.1016/j.devcel.2007.10.007 PMC2964935

[6] Rose L. & Gönczy P. Polarity establishment, asymmetric division and segregation of fate determinants in early C. elegans embryos. WormBook (2014). 10.1895/wormbook.1.30.2 25548889

[7] Arata Y., Lee J.-Y., Goldstein B. & Sawa H. Extracellular control of PAR protein localization during asymmetric cell division in the C. elegans embryo. Development 137, 3337–3345 (2010). 20823070 10.1242/dev.054742 PMC2934738

[8] Goehring N. W. Polarization of PAR proteins by advective triggering of a pattern-forming system. Science 334, 1137–1141 (2011). 22021673 10.1126/science.1208619

[9] Gross P. Guiding self-organized pattern formation in cell polarity establishment. Nat. Phys. 15, 293–300 (2019). 31327978 10.1038/s41567-018-0358-7 PMC6640039

[10] Motegi F. & Seydoux G. The PAR network: redundancy and robustness in a symmetry-breaking system. Philos. Trans. R. Soc. B Biol. Sci. 368, 20130010 (2013). 10.1098/rstb.2013.0010 PMC3785961 24062581

[11] Ng K. Cleavage furrow-directed cortical flows bias PAR polarization pathways to link cell polarity to cell division. Curr. Biol. 33, 4298–4311 (2023). 37729912 10.1016/j.cub.2023.08.076

[12] Koch L. A. & Rose L. S. Multiple pathways for reestablishing PAR polarity in C. elegans embryo. Dev. Biol. (2023). 10.1016/j.ydbio.2023.05.005 37263374

[13] Etemad-Moghadam B., Guo S. & Kemphues K. J. Asymmetrically distributed PAR-3 protein contributes to cell polarity and spindle alignment in early C. elegans embryos. Cell 83, 743–752 (1995). 8521491 10.1016/0092-8674(95)90187-6

[14] Gotta M., Abraham M. C. & Ahringer J. CDC-42 controls early cell polarity and spindle orientation in C. elegans. Curr. Biol. 11, 482–488 (2001). 11412997 10.1016/s0960-9822(01)00142-7

[15] Kumfer K. T. CGEF-1 and CHIN-1 regulate CDC-42 activity during asymmetric division in the Caenorhabditis elegans embryo. Mol. Biol. Cell 21, 266–277 (2010). 19923324 10.1091/mbc.E09-01-0060 PMC2808230

[16] Tabuse Y. Atypical protein kinase C cooperates with PAR-3 to establish embryonic polarity in Caenorhabditis elegans. Development 125, 3607–3614 (1998). 9716526 10.1242/dev.125.18.3607

[17] Watts J. L., Morton D. G., Bestman J. & Kemphues K. J. The C. elegans par-4 gene encodes a putative serine-threonine kinase required for establishing embryonic asymmetry. Development 127, 1467–1475 (2000). 10704392 10.1242/dev.127.7.1467

[18] Beatty A., Morton D. & Kemphues K. The C. elegans homolog of Drosophila Lethal giant larvae functions redundantly with PAR-2 to maintain polarity in the early embryo. Development 137, 3995–4004 (2010). 21041363 10.1242/dev.056028 PMC2976283

[19] Boyd L., Guo S., Levitan D., Stinchcomb D. T. & Kemphues K. J. PAR-2 is asymmetrically distributed and promotes association of P granules and PAR-1 with the cortex in C. elegans embryos. Development 122, 3075–3084 (1996). 8898221 10.1242/dev.122.10.3075

[20] Guo S. & Kemphues K. J. par-1, a gene required for establishing polarity in C. elegans embryos, encodes a putative Ser/Thr kinase that is asymmetrically distributed. Cell 81, 611–620 (1995). 7758115 10.1016/0092-8674(95)90082-9

[21] Hoege C. LGL can partition the cortex of one-cell Caenorhabditis elegans embryos into two domains. Curr. Biol. 20, 1296–1303 (2010). 20579886 10.1016/j.cub.2010.05.061

[22] Rodriguez J. aPKC cycles between functionally distinct PAR protein assemblies to drive cell polarity. Dev. Cell 42, 400–415. e9 (2017). 28781174 10.1016/j.devcel.2017.07.007 PMC5563072

[23] Sailer A., Anneken A., Li Y., Lee S. & Munro E. Dynamic opposition of clustered proteins stabilizes cortical polarity in the C. elegans zygote. Dev. Cell 35, 131–142 (2015). 26460948 10.1016/j.devcel.2015.09.006 PMC5963695

[24] Benton R. & St Johnston D. Drosophila PAR-1 and 14-3-3 inhibit Bazooka/PAR-3 to establish complementary cortical domains in polarized cells. Cell 115, 691–704 (2003). 14675534 10.1016/s0092-8674(03)00938-3

[25] Motegi F. Microtubules induce self-organization of polarized PAR domains in Caenorhabditis elegans zygotes. Nat. Cell Biol. 13, 1361–1367 (2011). 21983565 10.1038/ncb2354 PMC3208083

[26] Noatynska A., Tavernier N., Gotta M. & Pintard L. Coordinating cell polarity and cell cycle progression: what can we learn from flies and worms? Open Biol. 3, 130083 (2013). 23926048 10.1098/rsob.130083 PMC3758543

[27] Doerr S. & Ragkousi K. Cell polarity oscillations in mitotic epithelia. Curr. Opin. Genet. Dev. 57, 47–53 (2019). 31465986 10.1016/j.gde.2019.07.007

[28] Folkmann A. W. & Seydoux G. Spatial regulation of the polarity kinase PAR-1 by parallel inhibitory mechanisms. Development 146, dev171116 (2019). 30814118 10.1242/dev.171116 PMC6451319

[29] Morton D. G. The Caenorhabditis elegans par-5 gene encodes a 14-3-3 protein required for cellular asymmetry in the early embryo. Dev. Biol. 241, 47–58 (2002). 11784094 10.1006/dbio.2001.0489

[30] Ramanujam R., Han Z., Zhang Z., Kanchanawong P. & Motegi F. Establishment of the PAR-1 cortical gradient by the aPKC-PRBH circuit. Nat. Chem. Biol. 14, 917–927 (2018). 30177850 10.1038/s41589-018-0117-1

[31] Wu Y. & Griffin E. E. Regulation of cell polarity by PAR-1/MARK kinase. Curr. Top. Dev. Biol. 123, 365–397 (2017). 28236972 10.1016/bs.ctdb.2016.11.001 PMC5943083

[32] Bruinsma W. Inhibition of Polo-like kinase 1 during the DNA damage response is mediated through loss of Aurora A recruitment by Bora. Oncogene 36, 1840–1848 (2017). 27721411 10.1038/onc.2016.347 PMC5378932

[33] Gavet O. & Pines J. Progressive activation of CyclinB1-Cdk1 coordinates entry to mitosis. Dev. Cell 18, 533–543 (2010). 20412769 10.1016/j.devcel.2010.02.013 PMC3325599

[34] Macůrek L. Polo-like kinase-1 is activated by aurora A to promote checkpoint recovery. Nature 455, 119–123 (2008). 18615013 10.1038/nature07185

[35] Bishop A. C. A chemical switch for inhibitor-sensitive alleles of any protein kinase. nature 407, 395–401 (2000). 11014197 10.1038/35030148

[36] Lopez M. S., Kliegman J. I. & Shokat K. M. The logic and design of analog-sensitive kinases and their small molecule inhibitors. Methods Enzymol. 548, 189–213 (2014). 25399647 10.1016/B978-0-12-397918-6.00008-2

[37] Loyer N. A CDK1 phosphorylation site on Drosophila PAR-3 regulates neuroblast polarisation and sensory organ formation. eLife 13, e97902 (2024). 38869055 10.7554/eLife.97902 PMC11216751

[38] Michowski W. Cdk1 controls global epigenetic landscape in embryonic stem cells. Mol. Cell 78, 459–476 (2020). 32240602 10.1016/j.molcel.2020.03.010 PMC7214218

[39] Emptage R. P., Lemmon M. A. & Ferguson K. M. Molecular determinants of KA1 domain-mediated autoinhibition and phospholipid activation of MARK1 kinase. Biochem. J. 474, 385–398 (2017). 27879374 10.1042/BCJ20160792 PMC5317272

[40] Emptage R. P., Lemmon M. A., Ferguson K. M. & Marmorstein R. Structural basis for MARK1 kinase autoinhibition by its KA1 domain. Structure 26, 1137–1143 (2018). 30099988 10.1016/j.str.2018.05.008 PMC6092042

[41] Canagarajah B. Structural mechanism for lipid activation of the Rac-specific GAP, β2-chimaerin. Cell 119, 407–418 (2004). 15507211 10.1016/j.cell.2004.10.012

[42] Klinkert K. Aurora A depletion reveals centrosome-independent polarization mechanism in Caenorhabditis elegans. Elife 8, e44552 (2019). 30801250 10.7554/eLife.44552 PMC6417861

[43] Manzi N. I., de Jesus B. N., Shi Y. & Dickinson D. J. Temporally distinct roles of Aurora A in polarization of the C. elegans zygote. Development 151, dev202479 (2024). 38488018 10.1242/dev.202479 PMC11165718

[44] Munro E. M. PAR proteins and the cytoskeleton: a marriage of equals. Curr. Opin. Cell Biol. 18, 86–94 (2006). 16364625 10.1016/j.ceb.2005.12.007

[45] Nance J. & Zallen J. A. Elaborating polarity: PAR proteins and the cytoskeleton. Development 138, 799–809 (2011). 21303844 10.1242/dev.053538 PMC3035085

[46] Reich J. D. Regulated activation of the PAR polarity network ensures a timely and specific response to spatial cues. Curr. Biol. 29, 1911–1923. e5 (2019). 31155349 10.1016/j.cub.2019.04.058 PMC6584329

[47] Zhao P. Aurora-A breaks symmetry in contractile actomyosin networks independently of its role in centrosome maturation. Dev. Cell 48, 631–645 (2019). 30861375 10.1016/j.devcel.2019.02.012

[48] Schenk C., Bringmann H., Hyman A. A. & Cowan C. R. Cortical domain correction repositions the polarity boundary to match the cytokinesis furrow in C. elegans embryos. Development 137, 1743–1753 (2010). 20430749 10.1242/dev.040436 PMC3188577

[49] Liu J., Maduzia L. L., Shirayama M. & Mello C. C. NMY-2 maintains cellular asymmetry and cell boundaries, and promotes a SRC-dependent asymmetric cell division. Dev. Biol. 339, 366–373 (2010). 20059995 10.1016/j.ydbio.2009.12.041 PMC2903000

[50] Naganathan S. R. Morphogenetic degeneracies in the actomyosin cortex. Elife 7, e37677 (2018). 30346273 10.7554/eLife.37677 PMC6226289

[51] Swan K. A. cyk-1: a C. elegans FH gene required for a late step in embryonic cytokinesis. J. Cell Sci. 111, 2017–2027 (1998). 9645949 10.1242/jcs.111.14.2017

[52] Trong P. K., Nicola E. M., Goehring N. W., Kumar K. V. & Grill S. W. Parameter-space topology of models for cell polarity. New J. Phys. 16, 065009 (2014).

[53] Hubatsch L. A cell-size threshold limits cell polarity and asymmetric division potential. Nat. Phys. 15, 1078–1085 (2019). 10.1038/s41567-019-0601-x PMC6774796 31579399

[54] Nandan A. & Koseska A. Non-asymptotic transients away from steady states determine cellular responsiveness to dynamic spatial-temporal signals. PLOS Comput. Biol. 19, e1011388 (2023). 37578988 10.1371/journal.pcbi.1011388 PMC10449117

[55] Chang Y. & Dickinson D. J. A particle size threshold governs diffusion and segregation of PAR-3 during cell polarization. Cell Rep. 39, 110652 (2022). 35417695 10.1016/j.celrep.2022.110652 PMC9093022

[56] Illukkumbura R. Design principles for selective polarization of PAR proteins by cortical flows. Journal of Cell Biology vol. 222 (2023). 10.1083/jcb.202209111 PMC10238861 37265444

[57] Munro E., Nance J. & Priess J. R. Cortical flows powered by asymmetrical contraction transport PAR proteins to establish and maintain anterior-posterior polarity in the early C. elegans embryo. Dev. Cell 7, 413–424 (2004). 15363415 10.1016/j.devcel.2004.08.001

[58] Schierenberg E. Reversal of cellular polarity and early cell-cell interaction in the embryo of Caenorhabditis elegans. Dev. Biol. 122, 452–463 (1987). 3596018 10.1016/0012-1606(87)90309-5

[59] Featherstone C. & Russell P. Fission yeast p107 wee1 mitotic inhibitor is a tyrosine/serine kinase. Nature 349, 808–811 (1991). 1825699 10.1038/349808a0

[60] Lundgren K. mik1 and wee1 cooperate in the inhibitory tyrosine phosphorylation of cdc2. Cell 64, 1111–1122 (1991). 1706223 10.1016/0092-8674(91)90266-2

[61] Michael W. M. Cyclin CYB-3 controls both S-phase and mitosis and is asymmetrically distributed in the early C. elegans embryo. Development 143, 3119–3127 (2016). 27578178 10.1242/dev.141226 PMC5047676

[62] Russell P. & Nurse P. Negative regulation of mitosis by wee1+, a gene encoding a protein kinase homolog. Cell 49, 559–567 (1987). 3032459 10.1016/0092-8674(87)90458-2

[63] Milas A., Jagrić M., Martinčić J. & Tolić I. M. Optogenetic reversible knocksideways, laser ablation, and photoactivation on the mitotic spindle in human cells. in Methods in cell biology vol. 145 191–215 (Elsevier, 2018). 29957204 10.1016/bs.mcb.2018.03.024

[64] Robinson M. S., Sahlender D. A. & Foster S. D. Rapid inactivation of proteins by rapamycin-induced rerouting to mitochondria. Dev. Cell 18, 324–331 (2010). 20159602 10.1016/j.devcel.2009.12.015 PMC2845799

[65] Howell A. S. & Lew D. J. Morphogenesis and the cell cycle. Genetics 190, 51–77 (2012). 22219508 10.1534/genetics.111.128314 PMC3249366

[66] Treuner-Lange A. & Søgaard-Andersen L. Regulation of cell polarity in bacteria. J. Cell Biol. 206, 7–17 (2014). 25002676 10.1083/jcb.201403136 PMC4085708

[67] Devenport D., Oristian D., Heller E. & Fuchs E. Mitotic internalization of planar cell polarity proteins preserves tissue polarity. Nat. Cell Biol. 13, 893–902 (2011). 21743464 10.1038/ncb2284 PMC3149741

[68] Shrestha R. Mitotic control of planar cell polarity by polo-like kinase 1. Dev. Cell 33, 522–534 (2015). 26004507 10.1016/j.devcel.2015.03.024 PMC4464975

[69] Tio M., Udolph G., Yang X. & Chia W. cdc2 links the Drosophila cell cycle and asymmetric division machineries. Nature 409, 1063–1067 (2001). 11234018 10.1038/35059124

[70] McLellan M. M. Meru co-ordinates spindle orientation with cell polarity and cell cycle progression. EMBO J. 44, 2949–2975 (2025). 40169811 10.1038/s44318-025-00420-5 PMC12084343

[71] Swartz S. Z. Polarized Dishevelled dissolution and reassembly drives embryonic axis specification in sea star oocytes. Curr. Biol. 31, 5633–5641 (2021). 34739818 10.1016/j.cub.2021.10.022 PMC8692449

[72] Ragkousi K., Marr K., McKinney S., Ellington L. & Gibson M. C. Cell-cycle-coupled oscillations in apical polarity and intercellular contact maintain order in embryonic epithelia. Curr. Biol. 27, 1381–1386 (2017). 28457868 10.1016/j.cub.2017.03.064

[73] Jeyanathan G. Mitotic polarity oscillation promotes epithelial tumor progression. bioRxiv 2025–02 (2025).

[74] Das M. Oscillatory Dynamics of Cdc42 GTPase in the Control of Polarized Growth. Science 337, 239–243 (2012). 22604726 10.1126/science.1218377 PMC3681419

[75] Holmes W. R., Park J., Levchenko A. & Edelstein-Keshet L. A mathematical model coupling polarity signaling to cell adhesion explains diverse cell migration patterns. PLoS Comput. Biol. 13, e1005524 (2017). 28472054 10.1371/journal.pcbi.1005524 PMC5436877

[76] Meinhardt H. Orientation of chemotactic cells and growth cones: models and mechanisms. J. Cell Sci. 112, 2867–2874 (1999). 10444381 10.1242/jcs.112.17.2867

[77] Meinhardt H. & Gierer A. Applications of a theory of biological pattern formation based on lateral inhibition. J. Cell Sci. 15, 321–346 (1974). 4859215 10.1242/jcs.15.2.321

[78] Plazen L., Rahbani J. A., Brown C. M. & Khadra A. Polarity and mixed-mode oscillations may underlie different patterns of cellular migration. Sci. Rep. 13, 4223 (2023). 36918704 10.1038/s41598-023-31042-8 PMC10014943

[79] Jilkine A. & Edelstein-Keshet L. A comparison of mathematical models for polarization of single eukaryotic cells in response to guided cues. PLoS Comput. Biol. 7, e1001121 (2011). 21552548 10.1371/journal.pcbi.1001121 PMC3084230

[80] Town J. P. & Weiner O. D. Local negative feedback of Rac activity at the leading edge underlies a pilot pseudopod-like program for amoeboid cell guidance. PLoS Biol. 21, e3002307 (2023). 37747905 10.1371/journal.pbio.3002307 PMC10553818

[81] Wirtz-Peitz F., Nishimura T. & Knoblich J. A. Linking cell cycle to asymmetric division: Aurora-A phosphorylates the Par complex to regulate Numb localization. cell 135, 161–173 (2008). 18854163 10.1016/j.cell.2008.07.049 PMC2989779

[82] Loyer N. & Januschke J. Where does asymmetry come from? Illustrating principles of polarity and asymmetry establishment in Drosophila neuroblasts. Curr. Opin. Cell Biol. 62, 70–77 (2020). 31698250 10.1016/j.ceb.2019.07.018

[83] Oon C. H. & Prehoda K. E. Asymmetric recruitment and actin-dependent cortical flows drive the neuroblast polarity cycle. Elife 8, e45815 (2019). 31066675 10.7554/eLife.45815 PMC6524966

[84] Oon C. H. & Prehoda K. E. Phases of cortical actomyosin dynamics coupled to the neuroblast polarity cycle. Elife 10, e66574 (2021). 34779402 10.7554/eLife.66574 PMC8641948

[85] Bell G. P., Fletcher G. C., Brain R. & Thompson B. J. Aurora kinases phosphorylate Lgl to induce mitotic spindle orientation in Drosophila epithelia. Curr. Biol. 25, 61–68 (2015). 25484300 10.1016/j.cub.2014.10.052 PMC4291145

[86] Carvalho C. A., Moreira S., Ventura G., Sunkel C. E. & Morais-de-Sá E. Aurora A triggers Lgl cortical release during symmetric division to control planar spindle orientation. Curr. Biol. 25, 53–60 (2015). 25484294 10.1016/j.cub.2014.10.053

[87] Le Bras S. & Le Borgne R. Epithelial cell division–multiplying without losing touch. J. Cell Sci. 127, 5127–5137 (2014). 25344250 10.1242/jcs.151472

[88] Moreira S. PP1-mediated dephosphorylation of Lgl controls apical-basal polarity. Cell Rep. 26, 293–301 (2019). 30625311 10.1016/j.celrep.2018.12.060

[89] Osswald M. & Morais-de-Sa E. Dealing with apical–basal polarity and intercellular junctions: a multidimensional challenge for epithelial cell division. Curr. Opin. Cell Biol. 60, 75–83 (2019). 31153057 10.1016/j.ceb.2019.04.006

[90] Miller K. E., Kang P. J. & Park H.-O. Regulation of Cdc42 for polarized growth in budding yeast. Microb. Cell 7, 175 (2020). 32656257 10.15698/mic2020.07.722 PMC7328677

[91] Moran K. D. Cell-cycle control of cell polarity in yeast. J. Cell Biol. 218, 171–189 (2019). 30459262 10.1083/jcb.201806196 PMC6314536

[92] Witte K., Strickland D. & Glotzer M. Cell cycle entry triggers a switch between two modes of Cdc42 activation during yeast polarization. Elife 6, e26722 (2017). 28682236 10.7554/eLife.26722 PMC5536948

[93] Deutz L. N., Sarıkaya S. & Dickinson D. J. Membrane extraction in native lipid nanodiscs reveals dynamic regulation of Cdc42 complexes during cell polarization. Biophys. J (2023). 10.1016/j.bpj.2023.11.021 PMC11947473 38006206

[94] Dickinson D. J., Schwager F., Pintard L., Gotta M. & Goldstein B. A single-cell biochemistry approach reveals PAR complex dynamics during cell polarization. Dev. Cell 42, 416–434. e11 (2017). 28829947 10.1016/j.devcel.2017.07.024 PMC5575849

[95] Packer J. Atypical Protein Kinase C Promotes its own Asymmetric Localisation by Phosphorylating Cdc42 in the C. elegans zygote. BioRxiv 2023–10 (2024).

[96] Stiernagle T. Maintenance of C. elegans. (1999). 10.1895/wormbook.1.101.1 PMC4781397 18050451

[97] Arribere J. A. Efficient marker-free recovery of custom genetic modifications with CRISPR/Cas9 in Caenorhabditis elegans. Genetics 198, 837–846 (2014). 25161212 10.1534/genetics.114.169730 PMC4224173

[98] Dokshin G. A., Ghanta K. S., Piscopo K. M. & Mello C. C. Robust genome editing with short single-stranded and long, partially single-stranded DNA donors in Caenorhabditis elegans. Genetics 210, 781–787 (2018). 30213854 10.1534/genetics.118.301532 PMC6218216

[99] Kamath R. S. & Ahringer J. Genome-wide RNAi screening in Caenorhabditis elegans. Methods 30, 313–321 (2003). 12828945 10.1016/s1046-2023(03)00050-1

[100] Goehring N. W., Hoege C., Grill S. W. & Hyman A. A. PAR proteins diffuse freely across the anterior–posterior boundary in polarized C. elegans embryos. J. Cell Biol. 193, 583–594 (2011). 21518794 10.1083/jcb.201011094 PMC3087016

[101] Edgar L. G. & Goldstein B. Culture and manipulation of embryonic cells. in Methods in Cell Biology vol. 107 151–175 (Elsevier, 2012). 22226523 10.1016/B978-0-12-394620-1.00005-9 PMC3319665

[102] Hsu C. R., Xiong R. & Sugioka K. In vitro reconstitution of spatial cell contact patterns with isolated Caenorhabditis elegans embryo blastomeres and adhesive polystyrene beads. J. Vis. Exp. JoVE e60422 (2019). 10.3791/60422 31840666

[103] Shelton C. A. & Bowerman B. Time-dependent responses to glp-1-mediated inductions in early C. elegans embryos. Development 122, 2043–2050 (1996). 8681785 10.1242/dev.122.7.2043

[104] Ng K., Bland T., Hirani N. & Goehring N. W. An analog sensitive allele permits rapid and reversible chemical inhibition of PKC-3 activity in C. elegans. Micropublication Biol. 2022, (2022). 10.17912/micropub.biology.000610 PMC9391946 35996692

[105] Mori Y., Jilkine A. & Edelstein-Keshet L. Wave-pinning and cell polarity from a bistable reaction-diffusion system. Biophys. J. 94, 3684–3697 (2008). 18212014 10.1529/biophysj.107.120824 PMC2292363

[106] Dalwadi M. P. & Pearce P. Universal dynamics of biological pattern formation in spatio-temporal morphogen variations. Proc. R. Soc. A 479, 20220829 (2023).

[107] Han B. Polo-like kinase couples cytoplasmic protein gradients in the C. elegans zygote. Curr. Biol. 28, 60–69 (2018). 29276126 10.1016/j.cub.2017.11.048 PMC5763555

[108] Sells T. B. MLN8054 and Alisertib (MLN8237): Discovery of Selective Oral Aurora A Inhibitors. ACS Med. Chem. Lett. 6, 630–634 (2015). 10.1021/ml500409n PMC4468408 26101564

[109] De Henau S., Pagès-Gallego M., Pannekoek W.-J. & Dansen T. B. Mitochondria-derived H2O2 promotes symmetry breaking of the C. elegans zygote. Dev. Cell 53, 263–271 (2020). 32275886 10.1016/j.devcel.2020.03.008

[110] Fielmich L.-E. Optogenetic dissection of mitotic spindle positioning in vivo. Elife 7, e38198 (2018). 30109984 10.7554/eLife.38198 PMC6214656

